# Draft Genome Sequence of the Green Microalga *Chlorella* sp. Strain BAC9706, Isolated from Lake Baikal, Russia

**DOI:** 10.1128/MRA.00966-20

**Published:** 2020-10-22

**Authors:** Ivan S. Petrushin, Sergei I. Belikov, Olga I. Belykh, Irina Tikhonova, Lubov I. Chernogor

**Affiliations:** a Limnological Institute, Siberian Branch of the Russian Academy of Sciences, Irkutsk, Russia; b Irkutsk State University, Irkutsk, Russia; University of California, Riverside

## Abstract

Green algae of the phylum *Chlorophyta* are the most widespread autotrophic picoplankton in Lake Baika (Russia). To expand our molecular biological knowledge of these microalgae and compare them in the future with an endosymbiotic strain, we present here the draft genome sequence of *Chlorella* sp. strain BAC9706.

## Abstract (precis)

Green algae of the phylum *Chlorophyta* are the most widespread autotrophic picoplankton in Lake Baikal (Russia). To expand our molecular biological knowledge of these microalgae and compare them in the future with an endosymbiotic strain, we present here the draft genome sequence of *Chlorella* sp. strain BAC9706.

## ANNOUNCEMENT

Green algae of the phylum *Chlorophyta* are the most widespread autotrophic picoplankton in Lake Baikal (Russia) (1 – 3). The related algae are symbionts of freshwater sponges and were assigned to the genus *Choricystis* based on analysis of the 18S rRNA and *rbcL* genes (4, 5). To expand our molecular biological knowledge of these microalgae and compare them in the future with an endosymbiotic strain, we present a draft version of the genome sequence of the Baikal autotrophic picoplankton green alga *Chlorella* sp. strain BAC9706.

Water samples containing microalgae were collected from the pelagic zone in the southern part of Lake Baikal, 5 km from Listvyanka (51.47°38′N, 104.55°54′E). The pure strain was obtained by serial transfers on BG-11 medium-enriched agar plates. Individual colonies of algae were cultivated in f/2 medium in 0.5-liter spin flasks, under constant illumination (200 μE m^−2^ s^−1^ white fluorescent light). The dense culture was harvested by centrifugation (300 × *g*, 15 min); the pellet was rapidly transferred and stored at −80°C until DNA isolation. In this study, genomic DNA of the green microalga *Chlorella* sp. strain BAC9706 was extracted from microalgal cultures using the Xpert directXtract plant lysis kit (GRiSP, Portugal).

Whole-genome sequencing was performed using the MiSeq platform with paired-end chemistry (2 × 250 bp) (Illumina, USA). A DNA library was prepared from genomic DNA using a Nextera DNA Flex library prep kit (Illumina). A total of 12,987,796 paired-end reads was obtained, giving a coverage depth of 60×. The hypervariable region of the 18S rRNA gene sequence was assembled with GetOrganelle v. 1.6.2e (6) (with default settings). Strain BAC9706 showed the highest 18S rRNA gene phylogenetic affiliation to species of the phylum *Chlorophyta* belonging to the genus *Chlorella*.

A draft assembly was built using SPAdes v. 3.13.0 with default settings, raw read filtering, and error correction with a built-in BayesHammer module (quality threshold, 98%) (7). This draft assembly contained 5,837 contigs with an *N*_50_ value of 44,654 bp, and the largest contig was 317,606 bp long.

The resulting contigs were combined into scaffolds using Ragout v. 2.3 with default settings (https://github.com/fenderglass/Ragout) (8) using *Chlorella vulgaris* strain UTEX259 (GenBank accession no. VATW01000000) and *Chlorella vulgaris* strain UTEX 395 (GenBank accession no. LDKB01000000) as the reference genomes. We selected these two genomes from all the whole-genome sequences of *Chlorella* strains available in GenBank because the highest percentages of raw reads were mapped onto these genomes using Bowtie.

The final genome assembly had a total length of 32,957,766 bp, resulting in 201 scaffolds (*N*_50_, 521,038 bp; *L*_50_, 20), with a GC content of 61.92%; the largest scaffold was 1,817,640 bp.

Genome completeness analysis with BUSCO v. 3.1.0 using the “chlorophyta_odb10” data set with 2,168 benchmarking universal single-copy orthologs (BUSCOs) and default settings (9) showed results of 79.2% complete, 4.8% fragmented, and 16% missing. For the genome sequences of UTEX259 and UTEX 395, used as references, BUSCO reported 88.4% and 79.3% complete BUSCOs, respectively.

The presented draft nuclear genome assembly of *Chlorella* sp. strain BAC9706 provides a strong basis for comparative genetic analyses and will help elucidate metabolic processes.

### Data availability.

This whole-genome shotgun project has been deposited at DDBJ/ENA/GenBank under the accession no. JABWAE000000000. The raw reads are available via BioProject no. PRJNA591660 and SRA no. SRX7258937.
